# Association between soluble neprilysin and diabetes: Findings from a prospective longitudinal study

**DOI:** 10.3389/fendo.2023.1143590

**Published:** 2023-03-30

**Authors:** Junting Hu, Hanyun Zhu, Yunlang Dai, Yang Liu, Ying Lu, Shasha Zhu, Linan Chen, Mingzhi Zhang, Tingbo Jiang, Hao Peng

**Affiliations:** ^1^ Department of Cardiology, The First Affiliated Hospital of Soochow University, Suzhou, China; ^2^ Department of Epidemiology, School of Public Health, Medical College of Soochow University, Suzhou, China; ^3^ Jiangsu Key Laboratory of Preventive and Translational Medicine for Geriatric Diseases, Soochow University, Suzhou, China

**Keywords:** Chinese, diabetes, neprilysin, prospective longitudinal study, population epidemiology

## Abstract

**Background:**

The potential role of neprilysin (NEP) in glucose metabolism has been found by basic studies but lacks population evidence. The objective of this study was to examine the association between serum NEP and diabetes in Chinese adults.

**Methods:**

In a prospective longitudinal cohort study – the Gusu cohort (n=2,286, mean age: 52 years, 61.5% females), the cross-sectional, longitudinal, and prospective associations between serum NEP and diabetes were systemically examined by logistic regression adjusting for conventional risk factors. Serum NEP was measured at baseline using commercial ELISA assays. Fasting glucose was repeatedly measured 4 years apart.

**Results:**

The cross-sectional analysis found a positive association between serum NEP and fasting glucose at baseline (β=0.08, *P*=0.004 for log-transformed NEP). This association persisted after controlling for the dynamic risk profiles during follow-up (β=0.10, *P*=0.023 for log-transformed NEP). The prospective analysis found that a higher level of serum NEP at baseline was associated with a higher risk of diabetes during follow-up (OR=1.79, *P*=0.039 for log-transformed NEP).

**Conclusions:**

Serum NEP was not only associated with prevalent diabetes but also predicted the future risk of diabetes development in Chinese adults, independent of many behavioral and metabolic factors. Serum NEP may be a predictor and even a new therapeutic target for diabetes. However, the casualty and mechanisms of NEP in the development of diabetes require further investigation.

## Background

Neprilysin (NEP), a predominantly membrane-bound zinc-dependent type II metallopeptidase, is widely distributed in the body, including multiple tissues involved in glucose metabolisms, such as the liver, adipocytes, and pancreatic islets (1). By inactivating regulatory peptides *via* cleavage on the N-terminal side of hydrophobic residues, NEP is responsible for the breakdown of glucagon (2), and glucagon-like peptide 1 (GLP-1) (3), all of which play critical roles in glucose metabolism. These properties identified suggest a potential role of NEP in diabetes development and this hypothesis is also supported by findings from animal and human studies. For example, NEP deficiency inducted by gene knockout resulted in an increased islet β-cell mass and decreased glucose after 16 weeks of a high-fat diet in mice (4). Clinical trials found that NEP inhibition resulted in reduced hemoglobin A1c (HbA1c), fewer new-onset diabetes, and less insulin therapy in patients with diabetes (5) (6). Real-world studies also found that better glucose control were popular in patients with heart failure receiving the treatment of ARNi, a dual-acting angiotensin-receptor-neprilysin inhibitor (7). Of note, a considerable proportion of diabetic patients receiving ARNi did not get optimal glucose control (8), highlighting the unclear causality between NEP and diabetes. However, the association between circulating NEP and diabetes has been scarcely studied. A small clinical study found that urinary NEP was significantly increased in 20 patients with diabetes, compared to healthy controls (9). Another study including 144 patients with heart failure failed to observe a significant association between plasma NEP and HbA1c (10). The existing studies were mainly conducted in populations with European ancestry who have different risk profiles from Chinese. To date, no study has examined the association between circulating NEP and diabetes in Chinese population. Therefore, we aimed to examine the association between serum NEP and diabetes in a longitudinal cohort of Chinese adults in the Gusu cohort.

## Methods

### Participants

As detailed in the **Supplementary Data** (eMethods), the Gusu cohort was prospectively conducted in a traditional but economically developed district of Suzhou from January 2010 to December 2020. The study participants were randomly recruited by a cluster sampling procedure, with communities as the sampling unit. In 2010, eight communities were randomly selected as the research fields from the 39 communities in the Gusu district. All eligible participants residing in these fields were invited to participate if they were aged over 30 years, of Han ethnicity, and had lived in the area for at least 10 years. There were a total of 3,061 eligible residents in the study fields, but only 2,706 (participating rate: 88%) individuals agreed to participate in this study. After providing written informed consent, they received questionnaires and were offered free physical examination and clinical biochemical tests using blood and urine specimens under the principle of voluntary acceptance. Based on the information obtained, 208 participants were excluded from the cohort if they met at least one of the following criteria: (i) having clinical suspicion of diseases that may cause secondary hypertension (e.g., renal artery stenosis, coarctation, glomerulonephritis, pyelonephritis, pheochromocytoma, Cushing’s syndrome, Conn’s syndrome), (ii) self-reported history of CHD, stroke, or tumors, (iii) self-reported thyroid or parathyroid diseases, (iv) being pregnant, and (v) lacking blood samples. A total of 2,498 participants completed the baseline examination and were finally enrolled in the Gusu cohort study. Hereafter, all participants were followed every two years through 2020 for any CVD events and the survivors were invited to participate in the follow-up examination in 2014. The protocols of the Gusu cohort study were approved by the Soochow University Ethics Committee. Written informed consent was obtained from all study participants.

**Figure 1:**
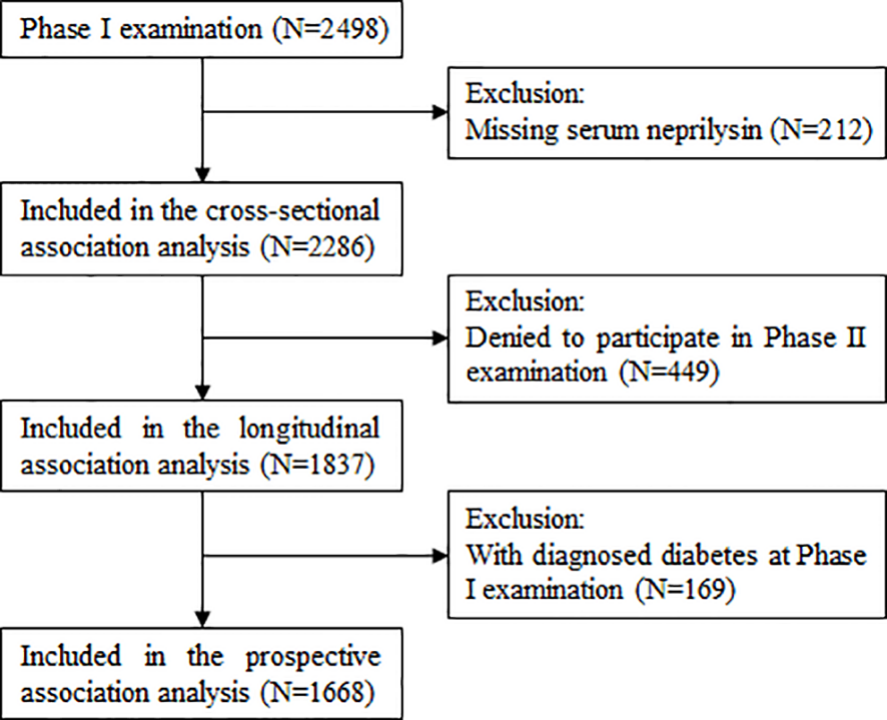
A flowchart illustrating the selection of participants and analytical plan.

**Figure 1** describes the selection of study participants for the current study. After excluding 212 participants with missing data on serum NEP, a total of 2286 participants were included in the cross-sectional association analysis. After further excluding 449 participants who refused to participate in the Phase II examination, 1837 participants were included in the longitudinal association analysis between serum NEP and dynamic fasting plasma glucose (FPG) during follow-up. Of these, 1668 participants free of diabetes at the Phase I examination were included in the analysis of the prospective association between baseline serum NEP and incident diabetes.

### Measurement of fasting plasma glucose and definition of incident diabetes

Venous blood was drawn in the morning after a requested overnight fast (at least 8 hours). FPG was measured immediately on Hitachi 7020 automatic biochemical analyzer using commercial reagents (Kangxiang Medical Appliances, Shanghai, PR of China). In our study, diabetes was defined as FPG ≥ 7.0 mmol/L or self-reported history of physician-diagnosed diabetes with a current prescription of insulin or oral hypoglycemic medications (11). Incident diabetes was defined as free of diabetes at baseline but initiated hypoglycemic medications during follow-up or with an FPG ≥ 7.0 mmol/L at the last follow-up examination.

### Measurement of serum NEP

The measurement of serum NEP was performed by skilled staff in the Jiangsu Key Laboratory of Preventive and Translational Medicine for Geriatric Diseases at Soochow University. Quantitative sandwich enzyme immunoassay (RayBiotech) was used to examine soluble NEP levels in serum samples stored at -80 °C. A standard curve was constructed and from which NEP concentrations of unknown samples were determined. Intra- and inter-assay coefficients of variation were less than 10% and 12%, respectively.

### Assessment of conventional risk factors

At the baseline examination, sociodemographic information (age, sex, and education level), lifestyle risk factors (cigarette smoking and alcohol drinking), and medical history were obtained by trained staff using standard questionnaires in the Chinese language. Metabolic factors (obesity, blood pressure, glucose, and lipids) were obtained by physical examination and laboratory testing. The detailed methods of data collection were presented elsewhere (12) and in the **Supplementary Data** (eMethods).

### Statistical analysis

All statistical analyses were conducted using R version 4.2.1. A two-tailed *P* value of less than 0.05 was considered statistically significant. Participants were divided into three groups according to tertiles of serum NEP. Their baseline characteristics were presented and compared across three groups. Serum NEP were log-transformed (log-NEP) to maximize the normality of data distribution and the generated values were used in downstream analyses.

### Cross-sectional analysis

To examine the association between serum NEP and prevalent diabetes, we first constructed a median regression model in which FPG at baseline was the dependent variable and log-NEP was the independent variable, adjusting for conventional risk factors including age, sex, education level, cigarette smoking, alcohol drinking, systolic blood pressure (SBP), body mass index (BMI), low-density lipoprotein cholesterol (LDL-C), high-density lipoprotein cholesterol (HDL-C), and hypoglycemic medication. Median regression was used here to account for the skewed distribution of FPG. We then similarly constructed a logistic regression model with prevalent diabetes (yes/no) as the dependent variable and serum NEP (log-NEP or categorical NEP in tertiles) as the independent variable.

### Longitudinal analysis

To examine whether serum NEP at baseline was associated with dynamic FPG during follow-up, we constructed a linear mixed regression model in which repeated measures of FPG were the dependent variable, serum NEP at baseline was the independent variable, adjusting for repeated measures of conventional risk factors listed above, with participants as the random effect. The mixed model was used here to account for repeated measurements and reduce the effects of dynamic risk profiles on FPG.

### Prospective analysis

To further examine whether serum NEP at baseline predicts the future risk of diabetes, we constructed a logistic regression model with diabetes at follow-up (yes/no) as the dependent variable and serum NEP at baseline was the independent variable, adjusting for covariates listed above as well as follow-up years. In this model, participants who had been already diagnosed with diabetes at baseline were excluded.

## Results

### Baseline characteristics of study participants

A total of 2286 participants (mean age: 52 years, 61.5% females) were included in the current study. Their baseline characteristics were shown in **Table 1**. Participants with a higher level of serum NEP were more likely to be drinkers and have a higher level of blood pressure and lipids but were less likely to be smokers (all *P*<0.05). No significant differences were found in the other variables listed.

**Table 1:** Baseline characteristics of study participants according to serum neprilysin levels.

Characteristics	Serum neprilysin, ng/mL			*P*-value for trend
	Tertile 1(~0.65)	Tertile 2(0.66~1.49)	Tertile 3(1.50~)	
No. of participants	764	759	763	–
Age, years	52.6 ± 9.3	52.0 ± 9.6	52.6 ± 9.0	0.995
Sex, males (%)	280(36.65)	307(40.45)	294(38.53)	0.449
Education, high school or above (%)	132(17.28)	173(22.79)	161(21.10)	0.064
Cigarette smoking, n (%)	188(24.61)	191(25.16)	153(20.05)	0.035
Alcohol drinking, n (%)	119(15.58)	150(19.76)	160(20.97)	0.007
Body mass index, kg/m^2^	24.75 ± 3.72	24.81 ± 3.62	24.89 ± 3.72	0.468
Systolic blood pressure, mmHg	129.0 ± 16.2	129.4 ± 16.4	131.6 ± 18.1	0.003
Diastolic blood pressure, mmHg	84.0 ± 8.8	84.9 ± 9.3	86.0 ± 9.5	<0.001
Total cholesterol, mmol/L	5.14 ± 1.11	5.19 ± 1.53	5.34 ± 2.47	0.030
Triglycerides, mmol/L	1.27 ± 0.92	1.54 ± 1.55	1.57 ± 1.85	<0.001
LDL cholesterol, mmol/L	3.01 ± 0.76	2.97 ± 0.75	3.03 ± 0.76	0.662
HDL cholesterol, mmol/L	1.52 ± 0.37	1.49 ± 0.50	1.52 ± 0.46	0.805

Results are expressed as mean ± SD (standard deviation) unless otherwise noted.

P-values for trend were tested by linear regression model for continuous variables and chi-square trend test for categorical variables. LDL, low-density lipoprotein; HDL, high-density lipoprotein.

### The cross-sectional association between serum NEP and diabetes

**Figure 2:**
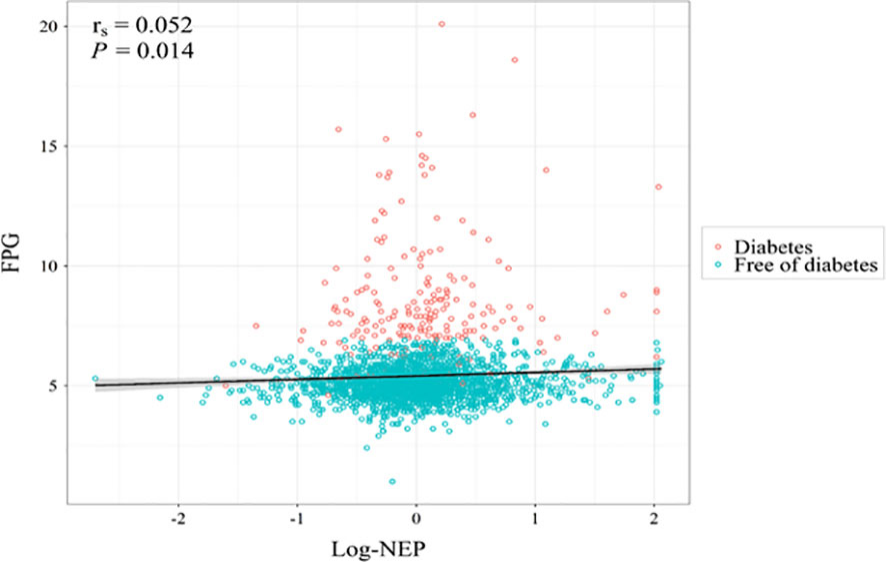
A scatterplot illustrating the association between serum neprilysin and fasting plasma glucose at baseline. Legend: The spearman correlation analysis found a significant correlation between serum neprilysin and fasting plasma glucose at baseline (r_s_=0.052, *P*=0.014). Red circles indicate individuals with diabetes and blue circles indicate individuals free of diabetes at baseline. After further adjustment for age, sex, education level, cigarette smoking, alcohol consumption, body mass index, systolic blood pressure, low- and high-density lipoprotein cholesterol, and hypoglycemic medication, the association was still significant (β=0.08, *P*=0.004). FPG, fasting plasma glucose; log-NEP, log-transformed serum neprilysin; r_s_, spearman correlation coefficient.

**Figure 3:**
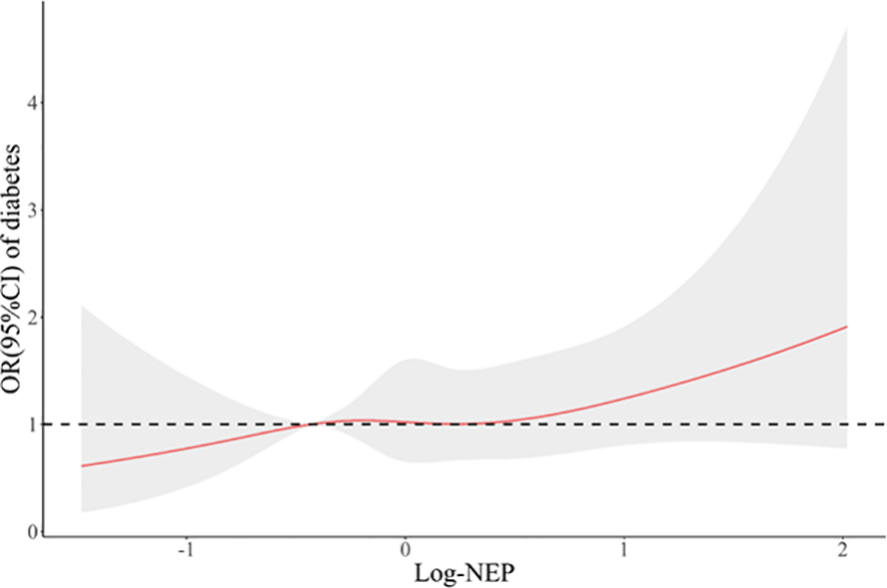
A restricted cubic spline curve illustrating the association between serum neprilysin and prevalent diabetes at baseline. Legend: A restricted cubic spline regression model was constructed with knots set at the 10^th^, 50^th^, and 90^th^ percentiles of the distribution of serum NEP. The red line indicates the odds ratios and the gray shadow indicates the corresponding 95% confidence intervals of prevalent diabetes in association with the increasing levels of log-NEP, compared with the midpoint of the 10^th^ percentile. Serum NEP was linearly (*P* for non-linearity=0.853) associated with a higher risk of prevalent diabetes (OR=1.28, *P*=0.072). OR, odds ratio; CI, confidence interval.

**Figure 2** displays a significant correlation between serum NEP and FPG at baseline (spearman r=0.052, *P*=0.014). The median regression found that after adjustment for conventional risk factors, serum NEP was still significantly associated with a higher level of FPG (β=0.08, *P*=0.004 for log-NEP). Logistic regression found that participants with a higher level of serum NEP were more likely to have diabetes (OR=1.28, *P*=0.072 for log-NEP, **Figure 3**).

### The longitudinal association between serum NEP and dynamic FPG

**Table 2** shows the association between baseline serum NEP and dynamic FPG during follow-up. The linear mixed regression found that a higher level of serum NEP at baseline was significantly associated with a higher level of FPG (β=0.11, *P*=0.018 for log-NEP). This association persisted after controlling for the dynamic risk profiles (β=0.10, *P*=0.023 for log-NEP). Compared to participants with the lowest level of serum NEP, those with the highest level of serum NEP had a 0.10 mmol/L increased level of PFG (β=0.10, *P*=0.076).

**Table 2:** The longitudinal association between baseline serum neprilysin and dynamic fasting plasma glucose during follow-up.

Serum neprilysin (ng/mL)	Unadjusted		Adjusted^*^	
	β(se)	*P*-value	β(se)	*P*-value
Log-NEP	0.11(0.05)	0.018	0.10(0.05)	0.023
Categorical				
Tertile 1	Reference	–	Reference	–
Tertile 2	0.04(0.07)	0.590	0.02(0.07)	0.709
Tertile 3	0.15(0.07)	0.027	0.10(0.06)	0.076

*Adjusting for sex, education level at baseline, and repeated measures of age, cigarette smoking, alcohol consumption, body mass index, systolic blood pressure, low-density lipoprotein cholesterol, high-density lipoprotein cholesterol at baseline and follow-up examinations.

### The prospective association between serum NEP and diabetes

In the follow-up period (mean 4.1 years, range 3.9-4.3 years), 100 of the 1668 participants free of diabetes at baseline developed new diabetes. The logistic regression found that a higher level of serum NEP at baseline was significantly associated with an increased risk of diabetes (OR=1.79, *P*=0.039 for log-NEP), independent of conventional risk factors. Compared to participants with the lowest level of serum NEP at baseline, those with the highest level of serum NEP had an 80% increased risk of diabetes during follow-up (OR=1.80, *P*=0.045, **Table 3**).

**Table 3:** The prospective association between serum neprilysin and incident diabetes during follow-up.

Serum neprilysin (ng/ml)	Incidence (%)	Unadjusted		Adjusted^*^	
		OR (95%CI)	*P*-value	OR (95%CI)	*P*-value
Log-NEP	100(5.23)	1.22(0.85-1.74)	0.276	1.26(0.85-1.85)	0.249
Categorical					
Tertile 1	24(3.74)	Reference		Reference	
Tertile 2	33(5.30)	1.67(0.96-2.97)	0.075	1.71(0.96-3.09)	0.071
Tertile 3	43(6.63)	1.79(1.04-3.15)	0.039	1.80(1.02-3.24)	0.045

^*^Adjusting for age, sex, education level, cigarette smoking, alcohol consumption, body mass index, systolic blood pressure, low-density lipoprotein cholesterol, high-density lipoprotein cholesterol at baseline and follow-up years.

## Discussion

In this large prospective cohort study, we found for the first time that serum NEP was not only associated with prevalent diabetes but also predicted an increased future risk of diabetes development in Chinese adults, independent of behavior and metabolic factors. Our results suggested that serum NEP could be a predictor and even a risk factor for diabetes.

In line with our study, the role of NEP in glucose metabolism has also been reported in previous studies (9, 13, 14). For example, an *in vitro* study found that NEP activity was upregulated in human dermal microvascular endothelial cells after short-term exposure to glucose (15). In animal experiments, *Nep* ^+/+^ mice displayed progressively elevated glucose levels over time, and *Nep^-/-^* mice exhibited improved glucose tolerance and elevated active GLP-1 levels (14). In humans, a small clinical study including 40 patients with diabetes complicated with chronic kidney disease and 20 healthy controls found that urinary NEP levels were significantly increased in patients with diabetes (9). A cross-sectional study including 318 white European males found that plasma NEP was elevated in patients with metabolic syndrome and associated with insulin resistance and obesity (16). However, these results are mainly generated from white populations and there is no evidence for the temporal association between NEP and incident diabetes, which is critical for the causal inference and clinical translation of NEP. In our study, we systemically examined the temporal relationship between serum NEP and diabetes in cross-sectional, longitudinal, and prospective aspects to rule out probable reverse causality. We found that a higher serum NEP at baseline was not only associated with higher prevalent diabetes but also predicted glucose elevation during follow-up and a higher future risk of incident diabetes. Together with prior studies, the consistent findings in our study increased the probability that elevated NEP may be a risk factor for diabetes. However, the causal association between serum NEP and diabetes still needs more evidence, from clinical trials in particular.

The mechanisms underlying the relationship between NEP and diabetes are not very clear and a better understanding of the mechanisms would improve the prevention and management of diabetes. There are several potential mechanisms through which NEP may participate in the regulation of glucose homeostasis. One possible mechanism could be the regulation of insulin secretion and glycemic homeostasis. NEP could be synthesized and expressed in islets (17), a target tissue of glucose metabolism. Inhibition of NEP could upregulate insulinotropic effects of incretin hormone GLP-1 (18) (19), suggesting the potential influence of NEP on insulin secretion. The second mechanism may be the contribution to insulin resistance. NEP perturbs insulin signaling and regulates the expression of the insulin receptor subunits in human subcutaneous white preadipocytes (20). NEP deficiency caused by gene knockdown could enhance insulin sensitivity and increase pancreatic β-cell function and mass in high-fat diet mice (4). Another possible mechanism may be the synergistic effects of NEP and its substrates. For example, the natriuretic peptide system also plays a crucial role in glucose metabolism (21) and the natriuretic peptides are mainly degraded by NEP. The complex role of NEP in glucose metabolism warranted further investigation to improve the precision medicine of diabetes. Recently, ARNi (sacubitril/valsartan), a compound preparation of NEP inhibitor accompanied with valsartan, has been recommended and widely used for patients with HFrEF (22). A *post-hoc* analysis from the PARADIGM trial (6) in which 3778 patients with known diabetes or previously undiagnosed diabetes with an HbA1c over 6.5% were randomized to receive either ARNi or enalapril found a greater reduction in HbA1c levels and new-onset diabetes in ARNi group compared to the enalapril group. In stark contrast, this superior blood glucose-control effect of ARNi was not significant in a real-world study in Korean patients (23). Due to the intimate interrelation between heart failure and diabetes, if NEP is proved to be one of the reliable biomarkers and core therapeutic targets for diabetes, killing two birds with one stone is undoubtedly a better treatment option. However, mechanisms through which NEP participates in diabetes need further investigation.

This study is the first to examine the prospective association between circulating NEP levels and diabetes risks in Chinese adults. The strengths included the prospective longitudinal study design, systemic analyses including cross-sectional, longitudinal, and prospective associations, and comprehensive assessment and adjustment for conventional risk factors including behavioral and metabolic factors. Some limitations should also be acknowledged. First, although some studies demonstrated a linear correlation between NEP expression and activity (24), it is still uncertain whether NEP levels in circulation perfectly correlate with biologically active tissue levels. We didn’t measure NEP activity, e.g., GLP-1, in this study, and further investigation of the relationship between serum NEP concentrations and activity is required. Second, as an observational study, residual confounding still exists in our study and the causal association between NEP and diabetes is still uncertain. Finally, given the lack of ethnic diversity, the generalization of our results to other ethnic populations should be cautious.

## Conclusions

Our study demonstrated that increased serum NEP was not only associated with prevalent diabetes at baseline but also predicted glucose elevation and an increased risk of diabetes development during follow-up in Chinese adults, independent of behavioral and metabolic factors. These results suggest that serum NEP may be a predictor and even a new therapeutic target for diabetes. Further investigation is urgently needed to illuminate the casualty and mechanisms of NEP in the development of diabetes.

## Data availability statement

Publicly available datasets were analyzed in this study. The datasets used and/or analyzed during the current study are available from the corresponding author on reasonable request.

## Ethics statement

The studies involving human participants were reviewed and approved by the Soochow University Ethics Committee. The patients/participants provided their written informed consent to participate in this study.

## Author contributions

TJ and HP conceived and designed the study. JH and HZ analyzed and interpreted the data. JH drafted the manuscript. HZ collaborated in writing of the methods. YD and YLiu contributed to the revision of the manuscript. SZ contributed to the interpretation of the results. YLu and LC assisted with the data collection and analysis. TJ and HP contributed to the interpretation of the results reviewed/edited of the manuscript and gave the final approval of the version to be published, and all authors agreed to be accountable for all aspects of the work. All authors contributed to the article and approved the submitted version.

## Supplementary material

Supplementary Data Sheet
**eMethods:** A detailed description of the methods of participants selection and data collection.
