# Supplementary material for: Association between soluble neprilysin and diabetes: Findings from a prospective longitudinal study
Source: Front Endocrinol (Lausanne). 2023 Mar 30;14:1143590. doi: 10.3389/fendo.2023.1143590 (PMC10100928; doi:10.3389/fendo.2023.1143590)
Supplement: Supplementary Data Sheet — eMethods: A detailed description of the methods of participants selection and data collection. [file DataSheet_1.docx]

**Supplementary data**

**eMethods**

**Participants of the Gusu cohort**

The Gusu cohort was a prospective study designed to identify new biomarkers for cardiovascular disease and its risk factors in Chinese adults. It was conducted in a traditional but economically developed district of Suzhou from January 2010 to December 2020. The study participants were randomly recruited by a cluster sampling procedure, with communities as the sampling unit. In 2010, eight communities were randomly selected as the research fields from the 39 communities in the Gusu district. All eligible participants residing in these fields were invited to participate if they were aged over 30 years, of Han ethnicity, and had lived in the area for at least 10 years. There were a total of 3,061 eligible residents in the study fields, but only 2,706 (participating rate: 88%) individuals agreed to participate in this study. After providing written informed consent, they received questionnaires and were offered free physical examination and clinical biochemical tests using blood and urine specimens under the principle of voluntary acceptance. Based on the information obtained, 208 participants were excluded from the cohort if they met at least one of the following criteria: (i) having clinical suspicion of diseases that may cause secondary hypertension (e.g., renal artery stenosis, coarctation, glomerulonephritis, pyelonephritis, pheochromocytoma, Cushing’s syndrome, Conn’s syndrome), (ii) self-reported history of CHD, stroke, or tumors, (iii) self-reported thyroid or parathyroid diseases, (iv) being pregnant, and (v) lacking blood samples. A total of 2,498 participants completed the baseline examination and were finally enrolled in the Gusu cohort study. Hereafter, all participants were followed every two years through 2020 for any CVD events and the survivors were invited to participate in the follow-up examination in 2014. The protocols of this study were approved by the Ethics Committee of Soochow University.

**Data collection at baseline**

Demographic data including age, sex, and education level were obtained by questionnaires administered by trained staff. Cigarette smoking was defined as current smoking or not. Current smoking was defined as having smoked at least 100 cigarettes in the entire life, having smoked cigarettes regularly, and smoking currently. Alcohol consumption was classified as current drinkers or not. Current drinkers were those who had consumed any alcoholic beverage ≥ 12 times during the past year. Three blood pressure measurements were performed by trained staff using a standard mercury sphygmomanometer and a cuff of appropriate size, according to a common protocol adapted from procedures recommended by the American Heart Association (1), after the participants had been resting for at least 5 min in a relaxed, sitting position. The first and fifth Korotkoff sounds were recorded as systolic blood pressure (SBP) and diastolic blood pressure (DBP), respectively. The means of the three measurements were used for statistical analyses. Body weight (kg) and height (cm) were measured when participants wore light clothes and no shoes by trained staff. Body mass index (BMI) was calculated by dividing weight in kilograms by the square of height in meters (kg/m^2^). Blood lipids including total cholesterol, triglycerides, high-density lipoprotein cholesterol (HDL-C), and low-density lipoprotein cholesterol (LDL-C), were measured by standard laboratory methods.

**Reference**:

1. Pickering TG, Hall JE, Appel LJ et al. Recommendations for blood pressure measurement in humans: an AHA scientific statement from the Council on High Blood Pressure Research Professional and Public Education Subcommittee. J Clin Hypertens (Greenwich) 2005;7:102-109.
